# Body image satisfaction and self-esteem status among the patients with polycystic ovary syndrome

**Published:** 2013-10

**Authors:** Fatemeh Bazarganipour, Saeide Ziaei, Ali Montazeri, Fatemeh Foroozanfard, Anoshirvan Kazemnejad, Soghrat Faghihzadeh

**Affiliations:** 1 *Department of Reproductive Health and Midwifery, Faculty of Medical Sciences, Tarbiat Modares University, Tehran, Iran.*; 2 *Mental Health Research Group, Health Metrics Research Center, Iranian Institute for Health Sciences Research, ACECR, Tehran, Iran.*; 3 *Department of Obstetrics and Gynecology, Kashan University of Medical Sciences, Kashan, Iran.*; 4 *Department of Biostatistics, Faculty of Medical Sciences, Tarbiat Modares University, Tehran, Iran.*; 5 *Faculty of Medical Sciences, Zanjan University of Medical Sciences, Zanjan, Iran*

**Keywords:** *Polycysticovarysyndrome*, *Self-esteem*, *Bodyimage*.

## Abstract

**Background:** Most previous research has focused on polycystic ovary syndrome (PCOS) characteristics and their association with psychological disorders, such as anxiety and depression.

**Objective: **In the present study, our aim was to study whether PCOS characteristics are associated with several aspects of psychological well-being namely self-esteem and body satisfaction.

**Materials and Methods:** This was a cross-sectional study of 300 women with PCOS that was carried out in Kashan, Iran. Main outcome measures were the Body Image Concern Inventory (BICI) and the Rosenberg’s Self-Esteem Scale and clinical information of PCOS. Major clinical PCOS features including obesity (BMI), excessive body hair (hirsutism score), acne, menstrual cycle disturbances and infertility.

**Results: **The findings of regression analysis indicated that infertile women had lower levels of self-esteem (=-0.11, p=0.049) and poorer body satisfaction (=0.121, p=0.036) compared with PCOS women without infertility. Furthermore, hirsute women experienced poorer self-esteem than women without hirsutism (=-0.124, p=0.032). Women with menstrual irregularities had higher body dissatisfaction (=0.159, p=0.005). Moreover, women with higher body mass index scores had poorer body satisfaction (=0.151, p=0.009) but were not associated with self-esteem.

**Conclusion:** The emotional well-being of the patients presenting with the syndrome needs to be recognized more fully, particularly in relation to the low self-esteem, poor body image, and struggles with weight, menstrual irregularities, hirsutism and infertility. The results of this study raise implications for clinical practice and suggest that a multidisciplinary approach to the management of women with PCOS.

This article extracted from Ph.D. thesis. (Fatemeh Bazarganipour)

## Introduction

Polycystic ovary syndrome (PCOS) is the most common endocrine disorders in women of reproductive age. It is estimated that 5-10% of women suffer from the disease (1). The burden of this condition on patient’ life and mental state is recognized, with studies revealing diminished quality of life and increased presence of depression symptoms among patient with PCOS, including suicidal ideation (2, 3). Some studies among patients with PCOS have indicated an impact on global self-esteem (4-6). 

It has been shown that women with PCOS have greater body dissatisfaction than healthy control women with regular cycles, even after adjustment for body mass index (BMI) (7, 8). Body image is defined as the mental picture of one’s body, an attitude about the physical self, appearance, and state of health, wholeness, normal functioning, and sexuality. Body image is a component of a larger concept of self that for women includes feeling feminine and attractive, enjoying one’s body as a symbol of social expression, and as a way of being in the world (9-12). The way in which one experiences her body is highly subjective, and is a product of her perceptions, thoughts, and feelings about body size, competence and function (12, 13). 

Negative perception of body image among PCOS include dissatisfaction with appearance, perceived loss of femininity, feeling less sexually attractive, and self-consciousness about appearance (14, 15). Experiencing high self-esteem may serve as a protective factor in coping with new and chronic illness, whereas low self-esteem is associated with anxiety, depression, and increased reports of general psychiatric (including somatic) symptoms (16). 

The association of self-esteem and depressive symptoms is known (17). Further, body dissatisfaction has been shown to predict the development of depressed mood and to be a risk and maintenance factor for the development of eating disorders (18, 19). Keeping in mind the importance that is placed on physical appearance in women in the majority of cultures, it is easy to understand why it is that women have the most problems when it comes to body image. Going a step further, for many women self-esteem is based exclusively on their body image and as a consequence their social functioning and interpersonal relations are affected (20). It becomes even more complicated when the woman suffers physical changes or disfigurement due to an illness such as PCOS. 

Changes in the appearance, irregular or absent menstrual periods, and difficulties in conceiving can result in psychological distress and may also influence the feminine identity of the patients with PCOS. Her perception of her physical appearance when compared to the society’s “ideal” might result in a negative impact on her emotional attitude and her quality of life. Today, the importance of addressing the prevention of body dissatisfaction as a public health issue is increasingly being recognized. In light of above considerations, it is likely that self-esteem and body image will reflect psychologically relevant consequences for the burden of PCOS for patients. 

Additionally, as above mentioned, body image and self-esteem develops in the context of socio-cultural factors and attitudes towards body for PCOS patients might varied by cultural issues. Research into body image and self- esteem has only recently extended to beyond women in western culture and related information from Iranian patient is very sparse, yet. In the present study, the principal focus was to study whether PCOS characteristics are associated with several aspects of psychological well-being namely self-esteem and body satisfaction. We hypothesized that all clinical characteristics are associated with psychological well-being. 

## Materials and methods

**Design and data collection**

This was a cross-sectional study of women with PCOS who attended two private gynecology clinics in Kashan, Iran from May to October 2012. Patients with confirmed diagnosis of PCOS were invited to participate in the study. After explaining the study objectives, written consent was obtained from each patient and they were requested to complete the study questionnaires. The Ethics Committee of the Tarbiat Modares University approved the study. This study was funded by grant of Tarbiat Modares University, Iran.

Inclusion criteria were: 15-40 years of age; married; Iranian; having two of the following Rotterdam diagnostic criteria: 1) Polycystic ovaries visualized on ultrasound scan (presence of 12 follicles or more in one or both ovaries and/or increased ovarian volume i.e., >10 ml), 2) clinical signs of hyperandrogenism (hirsutism score based on hirsutism score greater than 7 or obvious acne) and/or an elevated plasma testosterone (testosterone >2.0 nmol/l), 3) having an interval between menstrual periods >35 days and /or amenorrhea, defined as the absence of vaginal bleeding for at least 6 months (i.e.199 days) (20). 

Exclusion criteria were: having non-classic adrenal hyperplasia, thyroid dysfunction and hyperprolactinemia; having problems in speaking or listening; having of previous psychiatric diagnoses or using of psychiatric medications including antidepressants; taking any prescription medication (except allergy medications and occasional pain medications) for at least three months before entering the study.

**Measurement Instruments**

*Body image:* We used the Body Image Concern Inventory (BICI) to examine body image in our study. It contains 19 items related to dissatisfaction and concern about appearance, reassurance seeking, social concerns and avoidance related to appearance. For each item, respondents were asked to rate how often they had the described feeling or performed the described behavior on a Likert scale anchored by 1=‘‘never’’ and 5=‘‘always’’. The total score on the scale range from 19-95. The higher scores indicate higher dissatisfaction of body. Psychometric properties of the BICI in Iranian population have been verified (Table I) (21, 22). 

*Self-esteem*: The scale chosen to measure self-esteem was the Rosenberg´s Self-Esteem Scale (RSES), which employs 5-point Likert scaling. Respondents rate a series of 10 statements about themselves from ‘almost always true’ to ‘never true’. High scores indicate high self-esteem (16). Psychometric properties of the questionnaire in Iranian population have been verified (Table II) (23).

*Clinical and individual status*: include BMI, menstrual and infertility history, hirsutism, acne, age and socio-economic status.

- Menstrual history: interval between menstruation during preceding 12 months were asked and categorized into the following descending order in HRQOL status: 21-34 days; changeable; 35-60 days; <21 days,>199 days based on scores of HRQOL in our sample.
- Reproductive history: pregnancy history was asked and categorized into the following descending order in HRQOL status: has been pregnant: all births, no losses; has been pregnant: some births, some losses; never pregnant; has been pregnant: no births, all losses based on a previous study (24).
- BMI: weight and height were calculated by weight/height squared [kg/m^2^] in all patients.
- Body hair: clinical assessment of hirsutism was determined using the Ferriman-Gallwey Scoring System (F/G score). Nine body sites were graded from 0 (no terminal hair) to 4 (severe hirsutism). Scores can range from 0-36. A score of 7 or above was considered positive for hirsutism (25).
- Acne: acne was determined by the Global Acne Grading System (GAGS). The GAGS considers six locations on the face and chest or upper back, with a factor for each location based toughly on surface area, distribution, and density of pilosebaceous units.

The limits on the face are delineated by the hairline, jaw line, and ears. Each of the six locations is graded separately on 0-to-4 scale, with the *most *severe lesion within that location determining the local score. The global score is a summation of all local scores (26).

- Socio-demographic status: the study used years of formal education as a measure of socioeconomic status and it was categorized into five levels: no education, first level (1-5 years), second level (6-9 years), third level (10-12 years) and fourth level (more than 12 years). Studies from Iran showed that education could be a good proxy measure for socioeconomic status for Iranians (27).

**Statistical analysis**

Data are presented as number (%), unless otherwise indicated. Independent samples t-test and ANOVA was utilized for the comparison of the means of psychological variables between two or more groups based on PCOS characteristics, respectively. To explore the association between the PCOS characteristics and the psychological variables, the method of multiple linear regression analysis was applied. 

A stepwise approach was used to excluding factors that did not contribute significantly to the dependent variables. Independent variables included the PCOS characteristics that categorical variables were dichotomized and converted and coded as dummy variables. For example, menstrual cycle was converted to 1=having amenorrhea, oligomenorrhea, polymenorrhea or changeable; 0=the remaining. No multicollinearity problems were encountered when variance inflation factor (VIF) were analyzed. 

The normal distribution of the standardized residuals was examined graphically by normal probability plots and tested by the Kolmogorov-Smirnov test. Statistical analysis was performed using Statistical Package for the Social Sciences 15.0 (SPSS Inc., Chicago, IL, USA). P<0.05 was accepted as significant.

## Results

**The study sample**

Overall, 300 women with PCOS were included in the study during the six-month enrollment. The mean age of patients was 26.56 (SD=4.44) years. The majority of women had education beyond high school (72.7, n=218). More than two thirds of the patients had neither been pregnant nor had successfully carried a pregnancy to term and have abnormal menstruation. Socio-economic and clinical characteristic of the patients are presented in Table I. 

**Psychological well-being in patients**

Table II shows the mean scores for the psychological well-being based on PCOS characteristics. In relation to body image, it was found that patient with menstrual irregularities, infertility and obesity had more negative body image than subjects that were fertile, have normal menstrual or body weight. In relation to self-esteem, the women with more hirsutism or infertility had lower self-esteem than subjects without these symptoms.

For more thorough analysis of PCOS characteristics on the psychological well-being, we did multivariate linear regression (Table III). The findings of regression analysis indicated that infertile women had lower levels of self-esteem (=-0.11, p=0.049) and poorer body satisfaction (=0.121, p=0.036) compared with PCOS women without infertility. Furthermore, hirsute women experienced poorer self-esteem (=-0.124, p=0.032) than women without hirsutism. Women with menstrual irregularities had higher body dissatisfaction (=0.159, p=0.005) than normal menstruation. But, menstrual irregularities were not associated with self-esteem. Moreover, women with higher BMI scores had poorer body satisfaction (=0.151, p=0.009) but were not associated with self-esteem. 

**Table I:** Socioeconomic and clinical characteristic in PCOS patients

**Variable**		
Age (years)*		26.56 ± 4.44
Education**		
	The first level	32 (10.7)
	The second level	50 (16.7)
	The third level	126 (42)
	The fourth level	92 (30.7)
Hirsutism score*		6.7 ± 5.73
Acne score*		10.54 ± 7.26
Interval between menstruation (days)**		
	< 21	8 (2.7)
	21-34	109 (36.3)
	35-60	19 (6.3)
	>199	31 (10.3)
	Variable	133 (44.3)
Reproductive history**		
	Never pregnant	193 (64.3)
	Has been pregnant: all births, no losses	32 (10.7)
	Has been pregnant: some births, some losses	17 (5.7)
	Has been pregnant: no births, all losses	58 (19.3)
BMI (kg/m^2^)**		
	< 25	130 (43.3)
	25–30	120 (40)
	> 30	50 (16.7)

* Mean ±SD

** N (%)

**Table II:** Scores of psychological well-being baed on PCOS characteristics

**Variables**				**Self-esteem**	**Body image**
Hirsutism score					
			Normal F/G	29.54 ± 4.35	39.01 ± 14.31
			F/G>7	28.13 ± 3.86	39.05 ± 13.33
			p-value*	0.009	0.98
Acne					
		Mild		29.02 ± 4.15	39.27 ± 13.25
		Moderate		28.10 ± 3.20	39.64 ± 11.08
		Severe		31 ± 0.00	33.50 ± 9.81
		p-value**		0.31	0.66
Interval between menstruation (days) ^¥^					
		Normal		29.38 ± 4.07	35.78 ± 12.47
		Abnormal		28.96 ± 4.35	40.91 ± 13.76
		p-value*		0.41	0.001
Infertility					
	No			30.12 ± 4.34	37.82 ± 13.36
	Yes			28.85 ± 4.19	42.02 ± 13.54
	p-value*			0.03	0.014
BMI (kg/m^2^)					
	< 25			29.25 ± 4.19	36.52 ± 12.94
	25–30			29.09 ± 4.33	38.85 ± 12.14
	> 30			28.84 ± 4.26	46.10 ± 15.73
	p-value**			0.84	0.00

Data presented as mean (SD)

* student’s* t-*test

** ANOVA test

¥ Abnormal means having amenorrhea, oligomenorrhea, polymenorrhea or changeable; normal: the remaining

**Table III:** Multiple linear regression analysis of psychological well-being predictive in PCOS patients*

		**Coefficient ()**	**SE**	**p-value**	**95% CI**
Self-esteem					
	Hirsutism score	-0.124	0.042	0.032	-0.173 to -0.008
	Infertility	-0.113	0.601	0.049	-2.371 to -0.003
Body image					
	Infertility	0.121	0.134	0.036	0.545 to 0.943
	BMI	0.151	0.186	0.009	0.127 to 0.860
	Menstrual irregularities	0.159	1.58	0.005	1.333 to 7.570

* Hirsutism score, acne score and BMI were included in the regression analysis as continuous variables and other variables were used as dummy variables. Only significant results are presented.

## Discussion

Understanding body image is important to specify the social and psychological experience of being PCOS, the medical consequences of psychological issues, and the psychological contributors to the etiology of PCOS, but also to providing care. The goal of this study was to explore psychological well-being in women with PCOS in relation to certain PCOS characteristics. Results obtained in this research indicated that women with PCOS with menstrual irregularities had the greater body dissatisfaction.

It is imaginable that the absence of vaginal bleeding for a long period of time makes women feel insecure about their fertility as well as their femininity. Indeed, Keegan *et al *showed that women with PCOS associated regular menstruation and the capacity to bear children with femininity (28). Moreover, our results are in line with the findings of De Niet *et al* indicated that PCOS women with amenorrhea have lower levels of self-esteem and greater fear of negative appearance evaluation (29). 

Furthermore, the current study established that infertility was negatively associated with self-esteem and body satisfaction. For the infertile women, childlessness is an enormous psychological burden often associated with divorce, low social status and lowered self-perception because motherhood is perceived as an important part of female identity. A diminished sense of self-worth can develop, not only because her body has ‘failed’ but also because her self-esteem has been damaged (30). 

In some cultures, motherhood is the only way for women to enhance status in their family and community. The social pressure to have a child shortly after marriage is strong in the Iran. Current study findings are in general agreement with an earlier study by Winkvist and Akhtar (31). Many Islamic Pakistani women feel strongly that their childbearing pattern influences the way people treat them. They are more respected in the family when they have children (31, 32). Moreover, it was recently reported that infertile PCOS women had significantly higher depression scores and greater body dissatisfaction compared with the women with infertility from causes other than PCOS, which would also support the contribution of PCOS factors other than infertility (8).

Our results also indicate that BMI unfavorably affects body satisfaction. In relation to weight, poor body image in PCOS women may be compounded by cultural influences as it has been shown that android fat pattern is considered unattractive in many cultures (33). Previous studies have been shown that not only obesity acts as a risk factor for other health problems; it has been shown that obesity and weight gain are likely to lead to loss of self-esteem and poor body image, resulting in a decreased quality of life and psychological morbidity (34). 

Furthermore, the current study was shown that hirsutism was negatively associated with self-esteem. Likewise, Hahn *et al* found that hirsutism was negatively associated with sexual self-esteem and Benson *et al* findings indicated that the risk for clinically relevant depression was enhanced in patients who reported hirsutism (2, 35). In the same line with our results, half of the women with suspected PCOS in the study of Lipton *et al* felt that facial hair greatly affects their self-confidence and making them worry about their appearance (36). 

Contrastingly, Keegan *et al* did not report a difference in self-esteem between self-perceived hirsute and non-hirsute women (28). These differing results might be explained by the fact that the RSES is a general self-esteem questionnaire, which might not always be sensitive enough to measure fluctuations in self-esteem related to physical appearance. We don’t find a relationship between body satisfaction and self-perceived hirsutism in women with PCOS that in line with the previous study (28). In contrast, in De Niet *et al* study, women with hirsutism experienced poorer body satisfaction than without hirsutism (29). This difference in results might be explained by the fact that we used different criteria for hirsutism and different questionnaires to measure body satisfaction. 

Body dissatisfaction may be precipitating force underlying depression symptoms in women with PCOS. Stokes and Frederick- Recascino found happiness to be significantly and positively correlated to body image among non-PCOS women of reproductive ages (37). Several studies have reported that PCOS women have a worse body image than without PCOS. A theme among qualitative research among PCOS women is a lack of feeling feminine, and stigmatization related to PCOS symptoms (14). However, if these arguments are correct, then personal relationship variables would be expected to mediate the effects of body image and self-esteem on women’s well-being. Future studies are needed to test these hypotheses. Future studies are also needed to address some of the other limitations of the present study that are mentioned below. 

In particular, a more comprehensive measure of body image, which includes more than just body satisfaction, would allow a fuller exploration of the relationship between various aspects of body image and self-esteem. Prospective studies are needed to provide insight into the causal nature of the relationships between body image, and self-esteem, which cannot be satisfactorily addressed with correlational designs. 

However, it should be noted that it has certain limitations .The study of patients with PCOS who were attending two private gynecology clinics may limit generalization of the findings to the entire PCOS population. Also, patients referred to gynecology clinics may be different in certain socio-cultural and psychological variables compare to community. It is possible to suggest that majority of these patients have menstrual irregularities and infertility (not obesity and acne) because these patients with another complaints are often admitted to dermatology and endocrinology clinics. 

But, the demographic findings of present study have shown sufficient number of patient with these complaints that allow us to do this study. Moreover, all of the patients in this study were married. Therefore, the results of the present study have to be interpreted with some caution. In this study, absence non-PCOS controls matched on the various clinical features prevented the assessment of their individual contributions as principal influences on psychological well-being, separate from the diagnosis of PCOS. 

## Conclusion

The emotional well-being of the patients presenting with the syndrome needs to be recognized more fully, particularly in relation to the low self-esteem, poor body image, and struggles with weight, menstrual irregularities, hirsutism and infertility. The results of this study raise implications for clinical practice and suggest that a multidisciplinary approach to the management of women with PCOS.

## Conflict of interest

Not declared.
